# Depth perception between dots and the background face reduces trypophobic discomfort

**DOI:** 10.1186/s40359-022-01006-0

**Published:** 2022-12-06

**Authors:** Nanxin Song, Shinichi Koyama

**Affiliations:** Faculty of Art and Design, Graduate School of Comprehensive Human Sciences, University of Tsukuba, 1-1-1 Tennodai, Tsukuba, Ibaraki 305-8574 Japan

**Keywords:** Trypophobia, Perception of shadow and depth, Visual system, Visual discomfort, Dot pattern

## Abstract

**Background:**

Studies have shown that viewing a cluster of dots evokes feelings of discomfort in viewers and that the discomfort becomes especially strong when the dots are placed on background images of human skin. This phenomenon has been explained by the physical properties and spatial and semantic relationships between the dots and the background. However, it was not known whether the perceived, as well as the physical, spatial relationships contributes to the generation of discomfort.

**Methods:**

We evoked illusory depth perception between black dots and the background face by drawing shadow-like gray dots around the black dots, while keeping the same black dots at the same positions, and examined whether illusory depth perception could increase or decrease discomfort. In each trial, participants viewed one of the following types of facial images: (a) face-only (face without dots), (b) a cluster of black dots on the face, (c) a cluster of gray dots on the face, and (d) a cluster of black dots and shadow-like gray dots on the face. After seeing each picture, they evaluated how much discomfort they felt from viewing the picture using a Likert scale and reported whether they perceived depth between the dots and the face.

**Results:**

Participants felt discomfort toward all three types of faces with dots, that is, faces with black dots, gray dots, and both. However, interestingly, participants felt less discomfort when both black and gray dots were presented on the face than when only black dots were presented. The participants perceived depth between the black dots and the face in 85% of the trials with black dots and shadow-like gray dots, and there was a significant correlation between discomfort and frequency of depth perception. However, in the trials with black dots only and gray dots only, they perceived depth in only 18% and 27% of the trials, respectively, and the correlations between the frequencies of depth perception and discomfort were not significant.

**Conclusions:**

Our results suggest that the perceived spatial relationship, such as attached vs. separate, as well as the physical spatial relationship, contribute to the generation of discomfort.

## Background

Although dot patterns have been popularly used as motives in art and design (e.g., Yayoi Kusama [1]), they can evoke strong discomfort in viewers [2, 3]. Extremely strong discomfort is referred to as trypophobia [4]. The discomfort increases significantly when the dots are placed on the human skin [5, 6]. Dot patterns such as lotus seed pods on human skin are also used as motives by internet artists in Japan (e.g., Hasumaru [7], and these are widely known as HASU-COLLA (HASU = lotus seed pods; COLLA [i.e., collage] = photomontage image).

The generation of trypophobic discomfort is thought to involve both low-level visual processing and high-level visual processing. The involvement of low-level visual processing is indicated by the correlation between trypophobic discomfort and the physical characteristics of the dot pattern, such as spatial-frequency characteristics [4, 8, 9] and luminance changes (holes and bumps) of the dots [10]. On the other hand, the involvement of high-level visual processing is indicated by object recognition and the analysis of spatial relationships. Stronger discomfort can be evoked by the cluster consisting of disease-relevant elements, such as circular rash marks on a chest, as compared to those consisting of disease-irrelevant elements, such as drilled holes in a brick wall [11]. Discomfort became stronger when a cluster was placed on the human face than stones [5], thereby suggesting discomfort can be changed by the background and support such discomfort caused by reminding skin diseases [12–14]. Therefore, the strength of trypophobic discomfort depends not only on the physical characteristics of the dots per se but also on the semantic relationship between the dots and the background images.

**Fig. 1:**
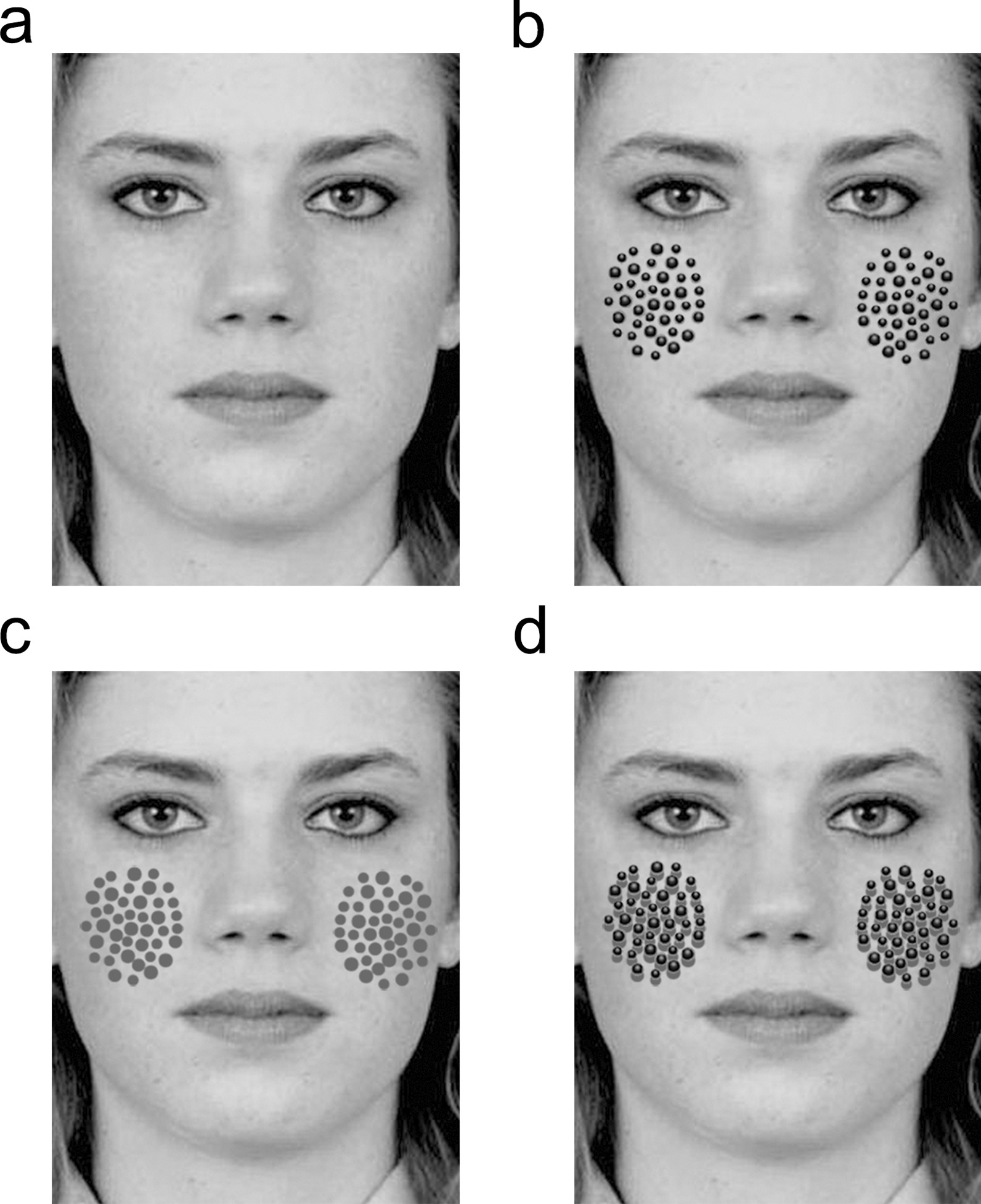
Four types of stimuli used in the discomfort evaluation: **a** Face-only, **b** Black dots on the face, **c** Gray dots on the face, and **d** Black dots and gray dots on the face. Permission for JACNeuf image [20] was obtained from Humintell/David Matsumoto

The spatial relationship between the dots and the background face also plays a role in the generation of discomfort. Viewers felt more discomfort toward dots on upright faces than on inverted faces [6]. However, it was still not clear how the perceived spatial relationship between the dots and the background face plays a role in the generation of discomfort. In previous studies, the dots were placed directly on background faces or objects, making them physically and perceptually attached to the background faces or objects. To separate the perceived spatial relationship from the physical relationship in the present study, we inserted shadow-like gray dots between the foreground black dots and the background face (see Fig. 1). Since the time of Leonardo da Vinci, it has been widely understood that cast shadows evoke a sense of depth [15–17]. If trypophobic discomfort simply depends on the number of dots, viewers would feel more discomfort toward a face that has both black dots and gray dots than they do toward a face with only black or gray dots. However, if the discomfort also depends on the perceived spatial relationship between the dots and face, we hypothesized that viewers would feel less discomfort toward the face with black dots and gray dots, as the presence of the gray dots allows the black dots to be perceived as separate from the face. We tested these hypotheses through a subjective discomfort rating experiment.

## Methods

### Participants

This study performed a one-way within-subject analysis of variance (ANOVA) using the difference between the three types of images as a within-participant factor. In order to detect an effect (α = 0.05, 1 − β = 0.80, *f* = 0.25) in a one-way within-subjects ANOVA, G*Power [18] estimated the sample size needed to be 28. Thus, 28 undergraduate and graduate students from the University of Tsukuba (17 females and 11 males; mean age = 24.6; SD = 0.6 years; range = 19–33 years) participated in this study. The average score of the 28 participants in the Japanese version of the Trypophobia Questionnaire (TQ-J) [10, 19], which measures trypophobia proneness, was 33.32 (SD = 14.52, range: 17–79). Among them, 10 participants (36%) scored higher than the cut-off value (= 31). All of the participants provided written informed consent. The experiment was conducted according to the principles of the Declaration of Helsinki and approved by the Ethics Committee of the Faculty of Art and Design, University of Tsukuba (Approval Number 29-11).

### Stimuli

Eight frontal facial images (two Japanese men and two Japanese women, two Caucasian men, and two Caucasian women) were taken from the Japanese and Caucasian Neutral Faces (JACNeuF) [20]. All facial images were converted to grayscale images. Using the eight pictures, we created four sets of stimuli (Fig. 1a–d). In the first set (Fig. 1a, face-only), we used eight grayscale pictures without adding dots. The average grayscale values were set to 140 in Adobe Photoshop CC2018, and the average luminance of the pictures was 100.6 cd/m^2^. In the second set (Fig. 1b, black dots on the face), a cluster of 42 dots was placed on each cheek of a face within an approximately 3.9° × 3.5° notional oval area. The dots were colored black and gray with circular gradation. The sizes and positions of each dot were based on a previous study [6] in which the dots were drawn by tracing real lotus seed pods. The gradation was added to enhance discomfort [10]. The 24 black dots were 0.3° in diameter, and the remaining 18 black dots were 0.4° in diameter. The average luminance of the black dots stimuli was 95.0 cd/m^2^, which was darker than the face-only stimuli, because of the presence of black dots on the face. In the third set (Fig. 1c, gray dots on the face), we took the position of black dots in the black dots stimuli as the coordinate axis, and the positions of the gray dots were shifted 0.1° left and 0.3° below on the left side of the face. On the contralateral side, the positions of the gray dots shifted 0.1° to the right and 0.3° below the right side of the face. Finally, we removed all the black dots and retained only the gray dots. The number of gray dots corresponds to the number of black dots in one set with black dots, for a total of 84. Gray dots were drawn with uniform gray, keeping the same sizes and shapes as the original black dots. The average luminance of gray dots stimuli was 95.7 cd/m^2^. In the fourth set (Fig. 1d, black dots and gray dots on the face), both black dots in the black dots stimuli and gray dots in the gray dots stimuli were presented together on the face. A cluster of black dots was presented on top of the cluster of gray dots. A cluster of 84 dots (42 black dots and 42 gray dots) was present on each side, and 168 dots (84 black dots and 84 gray dots) were placed on the face. The luminance of the black dots and gray dots stimuli was 93.3 cd/m^2^. In total, there were 32 stimulus images. The average luminance of the black dots was 6.0 cd/m^2^. The luminance of the gray dots (uniform gray) was 39.1 cd/m^2^. The average luminance of the face background around the dots was approximately 99.4 cd/m^2^. Therefore, the luminance contrast of the black dots on a face background was (99.4–6.0)/(99.4 + 6.0) = 0.89. The luminance contrast of the gray dots on a face background was (99.4–39.1)/(99.4 + 39.1) = 0.44. All images were subtended about 17.7° horizontally and 23.1° vertically and presented centrally on a white background on a 13-inch computer screen (MacBook Air, Model A1369).

**Fig. 2:**
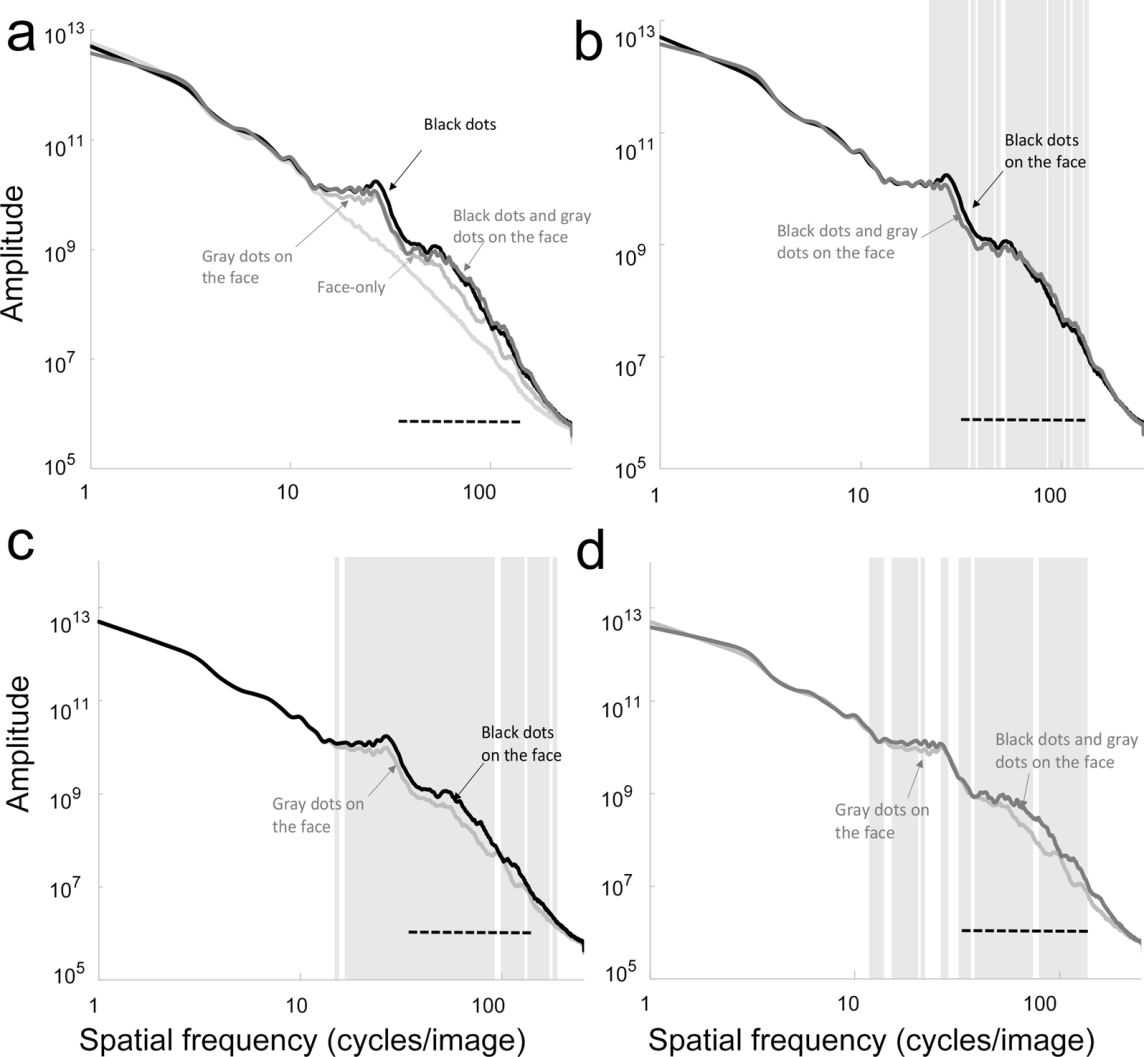
**a** Log–log plots of the average amplitude spectra in all types of the stimuli, i.e. Face-only, Black dots on the face, Gray dots on the face, and Black dots and gray dots on the face. **b** Log–log plots of the average amplitude spectra in Black dots on the face versus Black and gray dots on the face. **c** Log–log plots of the average amplitude spectra in Black dots on the face versus Gray dots on the face. **d** Log–log plots of the average amplitude spectra in Gray dots on the face versus Black and gray dots on the face. Gray zones in **b–d** indicate the spatial frequency bands at which there were significant differences between the two functions (*p* < .05). Dashed lines indicate the medium spatial frequencies (37.5–150 cycles per image) reported in a previous study [22]

Spatial frequency spectra of the four types of stimuli analyzed with the SHINE toolbox [21] were shown in Fig. 2. Contrast energies of each stimulus showed different patterns in the lower (37–60 cycles per image) vs. higher (67–150 cycles per image) ranges of the medium spatial frequencies (37.5–150 cycles per image) which were reported to contribute to discomfort [22]. In the lower (37–60 cycles per image) range, the contrast energy of the black dots was higher than those of black and gray dots, and gray dots. On the other hand, in the higher part (67–150 cycles per image), the contrast energy of the black and gray dots was higher than those of black dots and gray dots.

### Apparatus

The experiment was conducted in a lit room (approximately 1,000 lx). The participants rested on a comfortable chair, and the viewing distance was fixed at 57 cm away from the display. An Apple MacBook with a 13-inch, 1440 × 900 pixels display was used as the experimental apparatus, and the presentation of images was controlled using PsyScope X Program (available at http://psy.ck.sissa.it) [23] that ran presentation software and recorded participants’ evaluations.

### Procedure

**Fig. 3:**
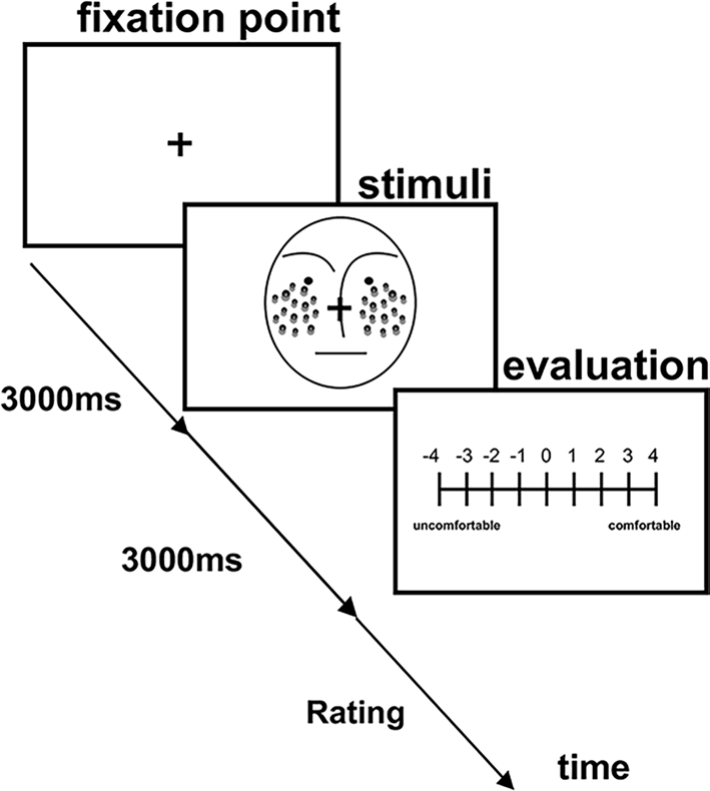
Sequence of events in a trial in the discomfort evaluation. Participants were asked to view the fixation point for three seconds. After showing the fixation point, a stimulus image was presented for three seconds. After that, an evaluation image was presented until the participant made a response

The experiment consisted of two types of evaluation tasks, a discomfort evaluation task and a depth evaluation task. In the discomfort evaluation task, each trial began with a central presentation of eye fixation (“ + ”) on a white background for three seconds (Fig. 3). The cross subtended about 1.0° horizontally and 1.0° vertically and was presented centrally on the white background of a computer screen. A facial stimulus followed, and one of the thirty-two images (consisting of eight face-only stimuli, eight black dots stimuli, eight gray dots stimuli, eight black dots and gray dots stimuli, respectively) was presented in random order for three seconds. After that, the participants were asked to evaluate the discomfort she/he felt from each image. An evaluation image was presented directly after each facial stimulus. Participants were asked to rate each image using a 9-point Likert scale, ranging from − 4 (uncomfortable) to 4 (comfortable), using the numeric keypad with unrestricted time. Each participant performed 32 trials: four image types (face-only stimuli, black dots stimuli, gray dots stimuli, black dots and gray dots stimuli) × eight facial images.

After the discomfort evaluation task, the participants underwent the depth evaluation task. The procedures were the same as the discomfort evaluation task, except that the participants were asked to judge whether they perceived depth between the foreground dots and the background face. After the facial stimulus was presented, an evaluation image with an evaluation scale was presented, and participants were asked to judge whether they felt the depth between black dots and the face (“yes” or “no”) using the numeric keypad. The viewing time and judgment time were unrestricted. Each participant performed 24 trials: three image types (black dots stimuli, gray dots stimuli, and black dots and gray dots stimuli) × eight facial images.

## Results

All 28 participants completed the discomfort evaluation task and depth evaluation task. In the discomfort evaluation task, the participants reported neutral comfort toward the face-only stimuli, whereas they reported negative comfort toward the other stimuli. One-sample two-tailed t-tests revealed that the discomfort rating scores of face-only stimuli were significantly larger than 0: *t*(27) = 2.35, *p* < 0.05, whereas the discomfort rating scores of the other three stimuli were significantly less than 0: black dots stimuli: *t*(27) = − 8.45, *p* < 0.01, gray dots stimuli: *t*(27) = − 4.76, *p* < 0.01, black dots and gray dots stimuli: *t*(27) = − 6.77, *p* < 0.01.

**Fig. 4:**
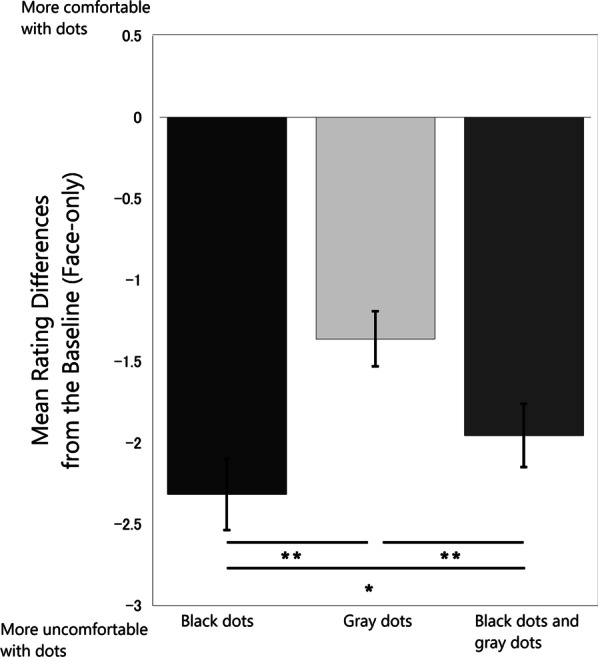
The results of the discomfort rating. Average scores of differential values of the baseline(face-only) and those in Black dots/ Gray dots/ Black dots and gray dots stimuli. The error bars indicate standard errors of the mean. **p < .01, *p < .05

In order to compare the effect of dots on the discomfort evaluation, we subtracted discomfort rating scores in the black dots stimuli, gray dots stimuli, and black dots and gray dots stimuli, respectively, from those in the baseline face-only stimuli for each subject. Figure 4 shows the mean rating differences of discomfort scores for the face-only images and the other stimuli with dots. A one-way within-subjects ANOVA showed differences in the mean rating of discomfort scores on the stimulus types, *F*(2,54) = 24.49, *p* < 0.01, η2 = 0.48. Bonferroni post hoc tests showed that the difference between the black dots stimuli and the face-only stimuli (black dots—face-only) was significantly greater than the between the black dots and gray dots stimuli and face-only stimuli (black dots and gray dots—face-only) (*p* < 0.05) and the difference between the gray dots stimuli and the face-only stimuli images (gray dots—face-only) (*p* < 0.01), respectively. The difference between the black dots and gray dots stimuli and face-only stimuli (black dots and gray dots—face-only) was significantly greater than that between the gray dots stimuli and face-only stimuli (gray dots—face-only) (*p* < 0.01).

The results of the depth evaluation task indicated that the participants perceived depth between the black dots and the background face at a high frequency when shadow-like gray dots were also included. The participants reported that they perceived depth in 84.82% of the trials with black dots and gray dots stimuli (*N* = 28), whereas they perceived depth in 18.30% of those with black dots stimuli (*N* = 28) and in 26.56% of those with gray dots stimuli (*N* = 24). Only 24 participants’ responses to the gray dots stimuli were analyzed because some response data were not recorded for four participants.

The results from correlation analysis between the frequencies of depth perception and discomfort evaluation scores indicated that the frequencies of depth perception with the black and gray dots stimuli had a significantly positive correlation with the discomfort evaluation scores (*r*[27] = 0.40, *p* < 0.05), indicating that participants who perceived the depth more frequently felt more comfortable with the stimulus. However, there were no significant correlations between the frequencies of the depth perception with the black dots on the face stimuli and the subsequent discomfort evaluation scores of each participant (*r*[27] = 0.22, *p* = 0.26), and the frequencies of the depth perception with the gray dot on the face stimuli and the average discomfort evaluation scores of each participant for the gray dots on the face stimuli (r[23] = − 0.28, *p* = 0.19).

The correlation between the contrast energies in the spatial frequency analysis and the discomfort evaluation was somewhat complicated. First, the results of the spatial frequency analysis were not consistent between the lower and higher ranges of the medium spatial frequencies (37.5–150 cycles per image), which were reported to contribute to discomfort [22]. Although contrast energies of the black dots on the face stimuli were higher than those of the gray dots on the face stimuli, and the black dots and gray dots on the face stimuli in the lower range (37–60 cycles per image); contrast energies of the black dots and gray dots on the face stimuli became higher than those of the black dots on the face stimuli and the gray dots on the face stimuli in the higher range (67–150 cycles per image). Second, the results from the spatial frequency analysis were not consistent with those from the discomfort evaluation. Although there was no significant difference between contrast energies between the black dots and gray dots on the face stimuli and the gray dots on the face stimuli in the lower range (37–60 cycles per image), discomfort evaluation for the black dots and gray dots on the face stimuli was stronger than for gray dots on the face stimuli. In the higher range (67–150 cycles per image), contrast energies of the black dots and gray dots on the face stimuli were higher than those for the black dots stimuli and gray dots stimuli, although the discomfort rating for the black dots and gray dots on the face stimuli was stronger than gray dots on the face stimuli and weaker than black dots on the face stimuli.

## Discussion

The present study demonstrated that the perceived spatial relationship between the foreground dots and the background face plays a significant role in the generation of discomfort. Discomfort is not simply depending on the number of dots, participants felt significantly less discomfort toward the black dots and gray dots stimuli. This is because in the black dots and gray dots stimuli, the black dots were perceived to be separate from the face and the gray dots were perceived as the cast shadow of the black dots, with depth perception between the dots and the face. The amount of discomfort depends on whether the dots are perceived as a part of the face or not, rather than simply on the number of dots. Based on our results, discomfort toward art and design, that contain dot patterns may be reduced by changing the grayscale contrast and/or the perceived spatial relationship between the dots and the background.

The results also support the hypothesis that our memory of skin diseases contributes to the generation of trypophobic discomfort [5, 6, 12–14, 24] because participants felt more discomfort when the dots were perceived as a part of the face. Participants’ discomfort was stronger in reaction to the black dots stimuli compared to the face-only stimuli, possibly because black dots as a visual cue might remind viewers of skin disease and/or pathogens. The discomfort was reduced when the shadow-like gray dots were drawn around the cluster of black dots because the dots seemed to be separated from the background face. This hypothesis was also supported by the correlation between the frequencies of the depth perception and discomfort evaluation scores in trials with black and gray dots on the face.

The results also suggest that the physical stimulus characteristics such as contrast energies in the lower medium spatial frequency ranges, and local luminance between the dots and the background faces might explain the discomfort to some extent. However, spatial frequency could not fully explain the results because there were some inconsistencies between the contrast energies and discomfort evaluation. Local contrast between the dots and the background faces could not explain the results fully either, because the black and gray dots stimulus had contrasts for both black and gray dots but the discomfort rating was not the strongest.

This study had some limitations. First, an illusory depth cue was used. Therefore, the effect of normal depth cues should be examined in a future study. Second, our results may be different in people with different sensory characteristics, such as those with sensory hypersensitivity and/or elderly people. Further studies will be necessary to better understand how dot patterns generate discomfort and how dots can be used more comfortably in art, design, and media.

## Conclusions

Perceived spatial relationship between a cluster of dots and the background face plays a role in the generation of discomfort. Our results suggest that perceived depth perception between the dots and the background face reduced discomfort, possibly because the dots do not seem to belong to the face. The results were consistent with those of previous studies showing that our memory of skin disease and pathogens contributes to trypophobic discomfort. Further studies will be necessary to better understand how dot patterns generate discomfort and how they should be used comfortably in art, design, and media.

## Data Availability

The stimuli and datasets used in the current study are available from the corresponding author upon reasonable request.
